# Save Your Eyes

**Published:** 1883-01

**Authors:** 


SAVE YOUR EYES.—We have had our paper made expressly for the INDEPENDENT PRACTITIONER. It is *fine cream laid* and free from glaze. Its color and comparatively non-reflecting surface greatly relieves the eye of the reader, which fact is of the utmost importance to the busy professional man who has to read at night and is dependent upon his keen vision for successful operations.

Our type is long primer, Roman face, and fresh from the type foundry, bought for publishing our journal. It makes the most legible print.

